# Supplementary material for: Twenty-Month Monitoring of Humoral Immune Response to BNT162b2 Vaccine: Antibody Kinetics, Breakthrough Infections, and Adverse Effects
Source: Vaccines (Basel). 2023 Oct 10;11(10):1578. doi: 10.3390/vaccines11101578 (PMC10611136; doi:10.3390/vaccines11101578)
Supplement: Supplementary file 1 [file vaccines-11-01578-s001.zip › vaccines-2628752-supplementary.pdf]

Table S1. IgG-NCP levels (bold positive results) at subsequent checkpoints for all individuals, by immune status ('vaccine only'/'hybrid' immunity), as well as by age (over/under 50 years).

Checkpoint					I	II	III	IV	V	VI	VII
Individuals			Age (y)	Gender [a]	**IgG-NCP (RU/ml) [b,*]**						
**Age < 50 years**	**Vaccine only**	1	37	F	0.03	0.41	**1.35***	**1.28**	0.03	**1.07***	**1.80***
		2	31	M	0.07	0.06	0.07	0.07	0.07	**2.46***	**2.92***
		3	47	F	0.07	0.08	0.07	0.07	0.07	**1.15***	0.97
		4	30	F	0.10	0.11	0.10	0.10	0.10	**4.10***	0.20
		5	44	F	0.37	0.34	0.37	0.37	0.37	**2.90***	0.57
		6	42	F	0.10	0.11	0.10	0.09	0.10	**2.80***	0.09
		7	47	F	0.15	0.34	0.15	0.12	0.15	**1.87***	ND
		8	47	F	0.10	0.11	**3.00***	**1.50**	0.10	0.20	0.06
		9	38	F	0.06	0.04	0.06	0.04	ND	0.25	**1.13***
		10	31	F	0.12	0.11	0.12	ND	0.12	0.17	**1.79***
		11	46	F	0.12	0.12	0.12	0.12	0.12	0.16	**1.76***
		12	47	M	0.07	0.09	0.07	0.09	0.07	0.14	**4.14***
		13	49	F	0.10	0.12	0.10	0.12	0.10	0.12	**1.86***
		14	46	F	0.09	0.09	0.09	0.09	0.09	0.10	**1.15***
		15	44	F	0.27	0.27	0.27	0.27	0.27	0.25	0.25
		16	30	M	0.17	0.16	0.17	0.16	0.17	0.24	0.40
		17	39	F	0.41	0.27	0.41	0.27	0.41	0.22	0.26
		18	26	F	0.11	0.12	0.11	0.12	0.11	0.14	0.07
		19	31	F	0.09	0.09	0.09	0.09	0.09	0.13	0.11
		20	29	F	0.01	0.08	0.01	0.08	0.01	0.11	0.46
		21	25	F	0.09	0.07	0.09	0.07	0.09	0.10	0.10
		22	34	F	0.07	0.06	0.07	0.06	0.07	0.08	0.20
		23	44	F	0.31	0.07	0.31	0.07	0.31	0.06	0.18
		24	35	F	0.03	0.12	0.03	0.12	0.03	0.04	0.16
		25	49	F	0.04	0.05	0.04	0.05	0.04	ND	0.04
		26	40	M	0.03	0.20	0.03	0.20	0.03	0.16	ND
		27	34	F	0.07	0.07	0.07	0.07	ND	0.13	ND
		28	26	F	0.01	0.16	0.01	0.16	0.01	0.10	ND
		29	31	F	0.06	0.06	0.06	0.06	0.06	0.08	ND
		30	42	F	0.64	0.41	0.64	0.41	0.64	0.07	ND
		31	26	F	0.58	0.27	0.58	0.27	0.58	ND	ND
		32	27	F	0.34	0.33	**2.70***	**1.20**	0.34	0.26	**1.23***
		33	32	M	0.04	**2.85**	**1.25**	0.84	ND	ND	ND
	**Hybrid immunity**	34	37	F	**3.52**	**2.75**	**2.17**	**1.58**	0.83	**5.32***	**2.17**
		35	41	F	**1.11**	0.65	0.55	0.45	0.07	**4.77***	**1.84**
		36	26	F	**4.69**	**3.61**	**2.51**	**1.02**	0.06	0.26	0.52
		37	33	F	**1.15**	0.72	0.52	0.12	0.07	0.08	0.09
		38	43	M	**1.41**	**1.12**	0.12	0.12	0.12	0.08	0.07
		39	27	M	**1.12**	0.81	0.51	0.21	0.05	ND	0.73
		40	47	F	**1.68**	**1.19**	0.75	0.04	0.20	0.70	0.58
		41	44	F	**1.96**	**1.30**	0.63	0.03	0.07	0.29	0.54
		42	46	F	**1.74**	0.70	0.15	0.07	0.16	0.11	0.37
		43	47	F	**1.71**	**1.13**	0.56	0.01	0.06	0.13	0.07
		44	44	F	**4.79**	**1.13**	0.06	0.06	0.41	0.06	0.06
		45	43	M	**5.55**	**5.05**	**5.18**	**4.63**	**3.25**	**2.28**	ND
		46	40	M	**1.73**	0.94	0.63	0.53	ND	ND	ND

Age ≥ 50 years	Vaccine only	47	65	M	0.07	0.07	0.07	0.07	0.07	**1.60***	**4.33***
		48	57	M	0.05	0.06	0.05	0.05	0.05	0.08	**4.06***
		49	56	F	0.05	0.06	0.05	0.05	0.05	0.09	**3.33***
		50	52	F	0.83	**2.86**	**1.56**	0.63	0.53	0.82	**2.74***
		51	60	F	0.69	0.68	0.69	0.69	0.69	0.53	**2.64***
		52	52	F	0.12	0.13	0.12	0.12	0.12	0.14	**1.88***
		53	57	F	0.05	0.05	0.05	0.05	0.05	0.06	**1.42***
		54	61	F	0.09	0.09	0.09	0.09	0.09	ND	0.71
		55	54	F	0.08	0.08	0.08	0.08	0.08	**1.48***	0.10
		56	50	M	0.06	0.06	0.06	0.06	0.06	0.07	0.86
		57	59	F	0.55	0.51	0.55	0.55	0.55	0.52	0.82
		58	55	F	0.03	0.22	0.03	0.03	0.03	0.32	0.82
		59	60	M	0.15	0.14	0.15	0.15	0.15	0.18	0.72
		60	51	F	0.06	0.06	0.06	0.06	0.06	0.09	0.65
		61	52	F	0.06	0.07	0.06	0.06	0.06	0.09	0.59
		62	59	F	0.12	0.10	0.12	0.12	0.12	0.19	0.47
		63	56	F	0.03	0.27	0.03	0.03	0.03	0.36	0.47
		64	60	F	0.04	0.08	0.04	0.04	0.04	0.11	0.41
		65	59	F	0.11	0.10	0.11	0.11	0.11	0.17	0.40
		66	53	F	0.04	0.04	0.04	0.04	0.04	0.09	0.26
		67	52	M	0.05	0.05	0.05	0.05	0.05	0.11	0.22
		68	61	F	0.06	0.06	0.06	0.06	0.06	0.10	0.19
		69	50	F	0.02	0.07	0.02	0.02	0.02	0.07	0.15
		70	54	F	0.09	0.08	0.09	0.09	0.09	0.10	0.13
		71	56	M	0.15	0.13	0.15	0.02	0.15	0.14	0.08
		72	57	F	0.07	0.10	0.07	0.15	0.07	0.09	0.06
		73	50	F	0.05	0.06	0.05	0.07	ND	0.05	0.04
		74	53	F	0.08	0.09	0.08	0.05	0.08	**1.35***	ND
		75	68	M	0.10	0.10	0.10	0.08	0.10	0.16	ND
		76	63	F	0.07	0.07	0.07	0.10	0.07	0.09	ND
		77	58	F	0.03	0.05	0.03	0.07	0.03	0.07	ND
		78	54	F	0.02	**2.31**	**1.54**	1.02	0.02	0.30	0.11
	Hybrid immunity	79	53	F	**6.37**	**5.65**	**4.70**	**3.15**	**2.25**	**1.72**	**4.54***
		80	55	F	**6.18**	**5.07**	**4.50**	**3.26**	**2.84**	**1.17**	**4.50***
		81	56	F	**7.20**	**7.12**	**7.00**	**5.63**	**4.60**	**4.12**	**2.75**
		82	50	F	**4.30**	**5.60***	**3.92**	**2.19**	**1.75**	**1.09**	0.45
		83	51	M	**1.22**	0.80	0.67	0.57	0.67	**5.38***	**3.51**
		84	67	M	**4.51**	**2.92**	**2.18**	**1.45**	**1.25**	**1.14**	0.44
		85	53	F	**1.82**	**2.84**	**1.43**	**1.17**	0.57	0.47	**3.68***
		86	59	F	**4.80**	**3.48**	**2.38**	**1.41**	0.77	0.87	0.55
		87	60	F	**1.14**	**4.23***	**3.16**	**1.52**	0.73	0.63	0.20
		88	64	F	**2.29**	**1.45**	**1.61**	**1.11**	0.75	0.85	0.39
		89	64	F	**1.17**	0.87	0.67	0.62	0.09	0.08	0.12
		90	65	F	**1.15**	**3.51***	**2.66**	**1.13**	0.38	0.28	ND
		91	80	M	**4.34**	**2.79**	**1.70**	**1.14**	0.75	**4.12***	**3.78**

[a] M, male; F, female.

[b] ND, no data.

* Results indicating breakthrough infections.
